# Hypertension in Asia 2021: A major contribution to worldwide understanding and management of hypertension

**DOI:** 10.1111/jch.14172

**Published:** 2021-01-17

**Authors:** Michael A. Weber, Daniel T. Lackland

**Affiliations:** ^1^ Division of Cardiovascular Medicine Downstate Medical College State University of New York Brooklyn NY USA; ^2^ Division of Translational Neurosciences and Population Studies Department of Neurology Medical University of South Carolina Charleston SC USA

The remarkable contributions of scientists in Asia to the field of hypertension continue at a rapid pace. It is only one year since the first issue dedicated to Hypertension in Asia was published in this journal, but exceptional advances in multiple areas of hypertension across Asia have compelled a 2021 edition.

When the previous 2020 issue was proposed and assembled, the two of us writing this brief editorial were then the editors of this journal and closely involved in supporting the creation of that work. But, since we are no longer officially connected to the journal, it is in our capacity as representatives of the World Hypertension League and as colleagues in hypertension research that we express our pleasure at the publication of this cutting‐edge collection of articles devoted to the clinical, public health and policy aspects of hypertension and its related conditions in Asia. The driving force behind this effort is the Asia HOPE Network whose creative work goes far beyond Asia and has a synergistic impact on research and clinical development across the world.

The collection of articles in this special issue includes systematic reviews, policy statements, and original work. There are two particularly important clinical practice statements put forward in this special publication: One deals with ambulatory blood pressure monitoring, and the other examines the relevance to Asia of recent practice guidelines written by expert committees in other parts of the world.

## 1 Blood pressure measurement

As far as blood pressure measurement is concerned, it is noteworthy that non‐invasive ambulatory blood pressure monitoring—mainly arm cuff‐based—has been available for almost 40 years, yet is still employed in only a tiny fraction of patients for the diagnosis or management of hypertension. Some reasons for the limited uptake of ambulatory monitoring are clear: The equipment is expensive; many patients find it cumbersome and intrusive during the 24‐hour monitoring period; and the time required for instructing patients, applying the equipment, and checking its accuracy represents a burden on busy practitioners. Still, research evidence has shown that ABPM values are more closely predictive of cardiovascular outcomes than office‐based blood pressure readings.^1^ Moreover, important early morning and nighttime blood pressure measurements are available only by this method, as are data predictive of cardiovascular outcomes such as short‐term blood pressure variability. ABPM is vital for understanding the duration–effects of hypertension therapies and so has become the standard measurement in hypertension clinical trials.

Even so, despite the multiple readings over the 24‐hour period provided by ABPM there is only modest reproducibility of data between consecutive monitoring periods in individual patients, presumably because it is difficult for patients to maintain consistent patterns of activity. The main reason for performing ABPM, as proposed by guideline statements, is to identify patients with either white‐coat hypertension, who perhaps do not require immediate treatment despite high office blood pressure measurements; and the important reverse situation of patients with “masked” hypertension characterized by office readings that are normal, but readings at home or elsewhere that are high. This condition is predictive of increased susceptibility to cardiovascular events.^2^ There is an alternative approach to check for masked hypertension: ask patients to perform self‐measurement at home with simple to use validated automated devices. On the other hand, new devices, several of which are not dependent on upper arm cuffs, may soon make whole‐day ABPM tolerable as well as inexpensive and acceptable to patients. Needless to say, it is in Asia that much of this eagerly awaited development is now underway.

Another blood pressure measurement issue addressed by a careful review in the Hypertension in Asia reports focuses on office blood pressure. All the recent guidelines have given considerable attention to essential features of this procedure. These include the correct posture of the patient; allowing the patient a short rest period before stating the measurement; using a validated automated oscillometric measuring device; taking 3 readings 1–2 minutes apart, and using the average of all 3 readings, or just the second and third, as the final blood pressure value; and not engaging the patient in conversation during the measurements. This careful technique largely eliminates the white‐coat effect although not the possibility of masked hypertension which is better addressed by patient self‐readings. The relevance and importance of developing this meticulous approach for office readings is carefully addressed in this issue.

## 2 Hypertension guidelines

Due to differences among countries, and even within countries, no single clinical practice guideline on hypertension can be universally applied. Nevertheless, recent major guidelines from the United States and from Europe have put forward very similar recommendations.^3^, ^4^ For instance, they have proposed similar office blood pressure treatment goals of 130/80 mmHg, albeit with a sharp difference in blood pressure targets for older patients—higher in Europe, but more aggressive and lower in the US. Choices of drug classes, the recommendation to initiate therapy with two agents from different drug classes and the use of single pill combinations of two or three drugs are very similar in both guidelines. Very recently, the International Society of Hypertension has written guidelines utilizing the evidence research performed by the US and European committees but then adapting the practice recommendations to more realistic and achievable goals in low‐ or medium‐resource settings.^5^ Most appropriately, the Hypertension in Asia project has taken a careful look at these differing guidelines. It is clear, however, that genetics and cultural, dietary, economic, and environmental factors in Asia often differ from those elsewhere, and so guidance must be adapted to address these factors. As well, there are major racial and ethnic variations among and within Asian countries and so ultimately each nation will need to develop its own relevant recommendations. The work reported in this special issue of the journal is an important step toward accomplishing this goal.

## 3 Hypertension and major co‐morbidities

Asian patients are particularly susceptible to strokes reflecting the high prevalence of hypertension in Asian communities, probably related to high salt intake among other factors. So, it is particularly appropriate that the HOPE Asia Network has included a detailed review on stroke in this special issue of the journal. Inevitably, secular changes in Asian communities, including the rapidly increasing consumption of high‐calorie and high‐fat fast foods, have affected cardiovascular outcomes as well. But stroke remains a conspicuous problem worldwide as well as in Asia. Recent guidelines on stroke prevention in the United States, while emphasizing tight blood pressure control for primary and secondary stroke prevention, acknowledge important questions that remain for optimizing immediate post‐stroke care.^6^

Our scientific colleagues in the HOPE Asia Network have addressed several other areas that have been newly recognized or where important progress has been made. For instance, the importance of sleep disorders is now recognized, particularly the common problem of obstructive sleep apnea that can strongly influence blood pressure. But even apparently common sleep patterns—either longer or shorter sleep periods than the recommended 7–9 hours duration in adults—can be associated with adverse cardiovascular outcomes. These topics, as well as an interesting consideration of the relationship between hypertension and mental health, are carefully considered in this publication. Chronic kidney disease, erectile dysfunction, and obesity are also expertly explored in the context of hypertension.

This issue of the journal is remarkable for its completeness. Indeed, it confronts important questions on hypertension treatment, including the frustrating clinical problem of poor patient adherence to prescribed treatment and the reluctance of clinicians and patients to adequately utilize dietary guidelines. Better understanding of the established hypertension drug classes, concepts such as the polypill (treating multiple cardiovascular risk factors with appropriate drugs combined into a single tablet) and the growing interest in applying artificial intelligence for managing hypertension are all covered here.

It is quite evident that much that is reported in this publication has important implications for hypertension experts across the globe. Yet, as we commented earlier, Asia is a unique continent with its own diversity of ethnicities, which leads to regional differences in clinical characteristics of hypertensive patients that should influence local clinical practice. Again, these areas are carefully considered in these pages along with unique therapies and interventions that have originated in Asia but should also be of interest to practitioners elsewhere.

## 4 A final comment

A publication project of the size and complexity of Hypertensin in Asia 2021 has demanded an exceptional effort by a very large number of leading hypertension experts across the entirety of Asia. It is impossible for us to adequately acknowledge the contributions of so many distinguished authorities. And so we respectfully ask our friend and colleague, Professor Kazuomi Kario from the Jichi Medical School in Japan, who has served as the deeply valued connection between the Asia HOPE Network and the World Hypertension League, to convey our warmest congratulations to the many clinical scholars who have contributed to this most powerful demonstration of Asian leadership in hypertension and cardiovascular disease.
